# The PPIase Activity of CypB Is Essential for the Activation of Both AKT/mTOR and XBP1s Signaling Pathways during the Differentiation of 3T3-L1 Preadipocytes

**DOI:** 10.3390/nu16152465

**Published:** 2024-07-29

**Authors:** Gyuhui Kim, Kyung-Sik Yoon, Joohun Ha, Insug Kang, Wonchae Choe

**Affiliations:** 1 Department of Biomedical Science, Graduate School, Kyung Hee University, Seoul 02447, Republic of Korea; eieiclo@khu.ac.kr (G.K.); sky9999@khu.ac.kr (K.-S.Y.); hajh@khu.ac.kr (J.H.); iskang@khu.ac.kr (I.K.); 2 Department of Biochemistry and Molecular Biology, School of Medicine, Kyung Hee University, Seoul 02447, Republic of Korea

**Keywords:** PPIase, CypB, 3T3-L1, adipocyte differentiation, AKT/mTOR, XBP1s

## Abstract

In this study, we undertook an extensive investigation to determine how CypB PPIase activity affects preadipocyte differentiation and lipid metabolism. Our findings revealed that inhibition of CypB’s PPIase activity suppressed the expression of crucial proteins involved in adipocyte differentiation and induced changes in proteins regulating the cell cycle. Furthermore, we clarified the impact of CypB’s PPIase activity on lipid metabolism via the AKT/mTOR signaling pathway. Additionally, we demonstrated the involvement of CypB’s PPIase activity in lipid metabolism through the XBP1s pathway. These discoveries offer invaluable insights for devising innovative therapeutic strategies aimed at treating and averting obesity and its related health complications. Targeting CypB’s PPIase activity may emerge as a promising avenue for addressing obesity-related conditions. Furthermore, our research opens up opportunities for creating new therapeutic strategies by enhancing our comprehension of the processes involved in cellular endoplasmic reticulum stress.

## 1. Introduction

Obesity represents a significant global health concern, exhibiting a marked upward trajectory in recent decades. As reported by the World Health Organization (WHO), approximately 13% of the global adult population was classified as obese in 2022 [1]. This metabolic disorder, characterized by a discrepancy between dietary intake and energy expenditure, results in the accumulation of excess adipose tissue, which increases the likelihood of developing a range of health complications, including but not limited to, cardiovascular diseases, type 2 diabetes, and cancer. Consequently, the prevention and treatment of obesity have emerged as a global public health priority [2]. While multiple interventions, including dietary modifications and physical exercise, are employed to treat obesity, they often yield only temporary effects. In addition, pharmacological and surgical interventions for obesity carry a potential for adverse effects, including psychiatric conditions such as depression, which could impede effective therapeutic outcomes [3]. Obesity is the result of the disordered deposition of fat droplets in adipocytes, leading to an expansion in white adipose tissue (WAT). This, in turn, elevates triglyceride (TG) content in liver and muscle tissues, causing pathological dysfunction [4]. Therefore, understanding the molecular mechanisms responsible for adipogenesis is crucial for developing effective strategies to prevent obesity and regulate lipid metabolism.

The cell line known as 3T3-L1 preadipocytes originates from the embryonic tissue of mice.

It serves as a central in vitro model for the study of adipogenesis and has garnered significant attention in obesity-related research [5]. This cell line is essential for elucidating the fundamental mechanisms of cell differentiation and lipid accumulation in adipose tissue, making it ideal for tracking changes in cell differentiation and adipocyte characteristics due to its high transfection efficiency and stability. The typical adipogenesis process in 3T3-L1 cells is composed of three distinct phases: release from contact inhibition, mitotic clonal expansion (MCE), and adipogenesis [6]. In the initial stage, contact inhibition triggers a halt in cell division, causing cells to remain in the G0/G1 phase of their growth cycle, after which differentiation is initiated using a differentiation medium containing MDI (dexamethasone, 3-isobutyl-1-methylxanthine (IBMX), and insulin). The process entails the exit of cells from the G1 phase, subsequent re-entry into the cell cycle, and the completion of multiple rounds of cell division, which represents a vital component of MCE [7]. Major transcription factors that regulate adipogenesis include CCAAT/enhancer-binding proteins (C/EBPs) and the peroxisome proliferator-activated receptor (PPAR) family [8]. During the MCE phase, early adipogenesis markers such as C/EBPβ and C/EBPδ are transiently elevated, subsequently promoting the activation of late adipogenesis markers like C/EBPα and peroxisome proliferator-activated receptor γ (PPARγ) [9]. These late-stage factors activate adipocyte-specific genes, such as adipocyte protein 2 (FABP4), ultimately leading to the mature adipocyte phenotype. This process is reported to involve the expression of cyclin-dependent kinase inhibitor, such as p27 [10,11]. Through the regulation of these critical transcription factors, lipid droplets containing neutral lipids appear in the cytoplasm, gradually enlarging and coalescing to complete adipocyte differentiation [12,13]. Consequently, many studies are focused on developing anti-obesity therapies by inhibiting adipogenesis or modulating adipogenic factors to control adipocyte differentiation.

The endoplasmic reticulum (ER) is a critical site for the synthesis of proteins, lipids, and sterols [14]. ER membrane-bound ribosomes release newly synthesized peptides into the ER lumen, where protein chaperones and foldases facilitate proper post-translational modification and folding [15]. Recent research has demonstrated, for the first time, that the unfolded protein response (UPR) is activated in the subcutaneous adipose tissue of individuals with obesity [16]. Within the unfolded protein response (UPR), one pathway includes IRE1α, an endoribonuclease situated in the membrane of the endoplasmic reticulum, which becomes active in response to ER stress. Upon activation, IRE1α splices XBP1 (X-box binding protein 1) mRNA, allowing the production of the active XBP1 protein [11]. XBP1, functioning as a transcriptional regulator, chiefly increases the production of chaperone proteins that aid in the folding of proteins inside the ER, thus improving the cell’s capacity to manage ER stress.

The activity of peptidyl-prolyl cis-trans isomerase (PPIase) is essential in the protein folding process as it facilitates the cis-trans conversion of proline residues, a key step in defining the structure and function of proteins [17]. PPIases are categorized into various protein families, including FK506-binding proteins (FKBPs), Cyclophilins (Cyps), and peptidyl-prolyl cis/trans isomerase NIMA-interacting 1 (Pin1), each of which is involved in multiple intracellular signaling pathways, particularly in adipogenesis [18,19,20]. FK506-binding protein 51 (FKBP51), belonging to the FKBP family, serves as a molecular chaperone involved in the cellular stress response and the folding of proteins. It has been reported that FKBP51 inhibits the Akt-p38 kinase pathway, thereby suppressing the glucocorticoid receptor alpha (GRα) while simultaneously activating peroxisome proliferator-activated receptor gamma (PPARγ), promoting adipogenesis [21]. Pin1 interacts with insulin receptor substrate-1 (IRS-1), enhancing insulin action and promoting adipogenesis. Pin1 acts as a key regulator of the insulin signaling pathway, and Pin1 knockout mouse models exhibit resistance to fatty diet-induced obesity, highlighting the important role of Pin1 in adipogenesis and obesity [22].

Cyclophilin, first recognized as the internal receptor for the immunosuppressive drug cyclosporin A (CsA), is extensively present throughout cellular structures [23]. One member of the Cyclophilin family, Cyclophilin B (CypB), also known as PPIB, is primarily found in the endoplasmic reticulum and nucleus, where it functions as a molecular chaperone with peptidyl-prolyl cis-trans isomerase activity, contributing to protein folding. Cyclophilins perform various physiological roles through these functions [24]. Additionally, they play critical roles in viral maturation and cellular proliferation pathways [25]. CypB has been implicated in the development of liver and gastric cancers [26]. It also participates in prolactin nuclear translocation to regulate human mammary epithelial and lymphocyte growth and differentiation [27]. Furthermore, in non-small-cell lung cancer, CypB regulates the signal transducer and activator of transcription-3 pathway, promoting cell proliferation, migration, invasion, and angiogenesis [28]. Recently we have demonstrated that CypB is involved in adipocyte differentiation [10,29]. In addition, Cyclophilins have been shown to possess chaperone-like activity, which effectively prevents the aggregation of various proteins, without requiring their peptidyl-prolyl isomerase (PPIase) activity. CypD has been demonstrated to affect a-synuclein aggregation independently of its PPIase activity [30]. Furthermore, PPIase-defective CypA has been shown to suppress aggression during arginine kinase refolding [31].

This study investigates the effects of inhibiting CypB PPIase activity on adipogenesis using the 3T3-L1 cell line. Owing to the crucial role of PPIase in protein folding, understanding the regulatory role of PPIases in protein folding can elucidate their molecular mechanisms and relevance to metabolic pathways associated with obesity [10,29]. Chronic inflammation, a hallmark of obesity, may be mitigated by the PPIase-mediated regulation of inflammation-related proteins. This regulatory role could potentially alleviate complications such as insulin resistance and cardiovascular diseases linked to obesity [32,33]. Furthermore, PPIases modulate cellular metabolism by regulating metabolic enzyme activity, suggesting innovative therapeutic approaches to rectify metabolic imbalances in obesity.

This study aims to provide supplementary therapeutic strategies for managing obesity. By investigating the impact of PPIase inhibition on adipogenesis, this research aims to complement existing treatments and facilitate the development of new strategies for managing complications associated with obesity.

## 2. Materials and Methods

### 2.1. Differentiation and Culture of Cells

3T3-L1 cells were grown in DMEM containing 10% calf serum and 1% penicillin/streptomycin at a temperature of 37 °C and a 5% CO_2_ environment until they were fully confluent. Upon reaching full confluence, the cells were further incubated for 2 additional days. Differentiation was then initiated by treating the cells with an MDI mixture (500 μM IBMX, 0.25 μM dexamethasone, and 10 μg/mL insulin) at 37 °C for a period of 48 h. After this treatment, the medium was replaced with DMEM supplemented with 10% fetal bovine serum and 1% penicillin/streptomycin, along with an additional 10 μg/mL of insulin, and the cells were maintained for another 2 days. From the fourth to the seventh day, the medium was refreshed continuously to sustain the differentiation process. On the seventh day, the cells undergoing differentiation were examined under a light microscope at 40× magnification.

### 2.2. Plasmid and Transfection

Initially, 3T3-L1 cells were plated at densities of 5.5 × 10^5^ cells/mL in 60 mm dishes or 12 × 10^5^ cells/mL in 100 mm dishes and were incubated for 24 h at 37 °C in an environment with 5% CO_2_. This was followed by plasmid transfection using X-tremeGene^®^ HP DNA transfection reagent (MilliporeSigma, St. Louis, MO, USA), adhering to the manufacturer’s instructions, and then the cells were incubated for another 48 h under the same conditions. After two days, further experiments were conducted. The cDNA encoding the C-terminal portion of 216 amino acids of wild-type CypB and the PPIase-deficient CypB R95A mutant was cloned into the pcDNA vector (Invitrogen, Waltham, MA, USA), between the HindIII and EcoRV restriction sites, to enable protein expression [34]. An HA tag was also added to the C-terminus to produce the protein with the HA tag sequence [10].

### 2.3. Western Blot Analysis

Proteins from cells were extracted using RIPA buffer, which includes 50 mM Tris-HCl at pH 7.6, 150 mM NaCl, and 1% each of Triton X-100 and sodium deoxycholate. The protein levels were measured with the Bradford method. A consistent quantity of protein (20 μg) was run through a 6–15% SDS-PAGE gel and then moved onto a BioTrace NT nitrocellulose membrane (Pall Life Sciences, Port Washington, MA, USA). This membrane was then treated with a 5% blocking agent (either BSA from GendEPOT, LLC or skim milk from MB cell) for one hour at ambient temperature. Afterward, the membrane was incubated with the primary antibody at 4 °C overnight. A corresponding secondary antibody was then applied, and the incubation continued at room temperature for an hour. The dilutions for the primary and secondary antibodies were 1:1000 and 1:10,000, respectively. Bands were detected using Clarity Western ECL Substrate (Bio-Rad Laboratories, Inc., Hercules, CA, USA), and their density and size were analyzed with ImageJ software (v1.54i), with normalization to Vinculin and β-actin. Antibodies targeting Vinculin(sc-25336), FABP4(sc-18661), C/EBPβ(sc-7962), β-actin(sc-47778), GRP78(sc-1050), CyclinA(sc-271645), CyclinD(sc-8396), p27(sc-1641), IRE1α(sc-31199), and SREBP1(sc-13551) were sourced from Santa Cruz Biotechnology (Santa Cruz, CA, USA). Meanwhile, antibodies for HA(C29F4), PPARγ(2443S), C/EBPα(2295S), XBP1s(83418S), CDK4(12790S), p-RB(8516S), p-mTOR(5536S), p-p70S6K(9208S), p70S6K(9202SL), ACC(3662S), FAS(3180S), mTOR(2983S), AKT(5298M), and p-AKT(4060S) were obtained from Cell Signaling Technology (Danvers, MA, USA).

### 2.4. Oil-Red-O Staining

After a week of differentiation, the 3T3-L1 cells underwent three washes with phosphate-buffered saline (PBS). They were then fixed using 3.7% paraformaldehyde for one hour at ambient temperature and washed three more times with PBS. Subsequently, the cells were dyed with 0.5% Oil Red O solution in isopropanol for an hour at room temperature. After staining, the cells were rinsed thrice with pure water. The dyed cells were then examined under a brightfield microscope (Olympus Corporation’s model IX73, Tokyo, Japan) at a magnification of 100×, with five distinct random fields documented for each well. The cells, once stained, were immersed in 500 μL of isopropanol at ambient temperature for a duration of 10 min. The lipid droplets were then quantified at a wavelength of 510 nm using a microplate reader from Molecular Devices, LLC (San Jose, CA, USA).

### 2.5. FACS Analysis

3T3-L1 cells were plated at a density of 1 × 10^5^ cells per dish in 60 mm dishes and allowed to grow. Once confluence was achieved, the cells were incubated in a medium containing agents that induce differentiation, such as MDI. Following a 24 h period, the cells were collected, cleansed with PBS, fixed in 70% ethanol, and then stained with Muse™ Cell Cycle Reagent (Luminex Corporation, Austin, TX, USA) for half an hour. The different phases of the cell cycle were then examined using the Muse™ Cell Analyzer.

### 2.6. Statistical Analyses

The results were presented as the average value plus or minus the standard deviation from three separate experiments, except where noted otherwise. The data were analyzed using a one-way ANOVA followed by Tukey’s test for multiple comparisons, and the differences between two groups were evaluated using a two-tailed Student’s *t*-test without pairing. Results were deemed to be statistically significant if the *p*-value was less than 0.001.

## 3. Results

### 3.1. Impact of CypB PPIase on 3T3-L1 Cell Differentiation

Previous studies have shown that elevated levels of CypB enhance the differentiation of adipocytes [10]. To investigate the influence of PPIase activity on adipogenesis, 3T3-L1 preadipocytes were treated with an adipogenic inducer, MDI (a mixture of methylisobutylxanthine, dexamethasone, and insulin), over a period of seven days.

**Figure 1:**
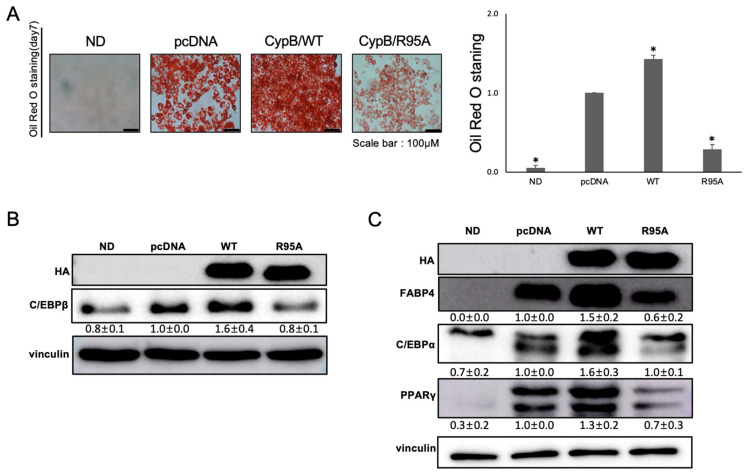
Influence of CypB PPIase activity on adipogenesis in 3T3-L1 cells. When 3T3-L1 cells reached 70–80% confluence, they were transfected with pcDNA, CypB/WT-HA, or CypB/R95A and incubated at 37 °C for 48 h. The cells were then permitted to undergo differentiation from day 2 to day 7. (**A**) To assess lipid storage, differentiated cells (ND, pcDNA, CypB/WT, and CypB/R95A) underwent Oil Red staining. The quantity of cytoplasmic lipid droplets was determined by measuring the absorbance at 510 nm. (**B**) Two days after initiating adipogenesis, the expression levels of C/EBPβ, a key regulator in early adipogenesis, were analyzed through Western blot, using β-actin as the loading control. (**C**) Seven days after the start of adipogenesis, the expression of late-stage adipogenic markers (C/EBPα, PPARγ, and FABP4) was measured via Western blot, with vinculin as the loading control. The displayed result is typical of three separate experiments that were performed in triplicate wells. Error bars indicate the mean ± standard deviation; * *p* < 0.001 signifies statistical significance.

Lipid accumulation was then observed using Oil Red O staining to detect intracellular triglycerides. The results showed that lipid accumulation in CypB/R95A cells, which lack PPIase activity, was reduced to approximately 20% of the level observed in CypB/WT cells. Additionally, lipid accumulation in CypB/R95A cells was significantly lower than in the control cells transfected with pcDNA (Figure 1A). Preadipocytes differentiate into adipocytes through the complex interplay of differentiation regulators expressed at early, mid, and late stages. Early induced C/EBPβ plays a critical role in regulating the expression of mid-stage and late-stage factors C/EBPα and PPARγ, which are essential for adipocyte differentiation. When confluent preadipocytes are treated with MDI, this stimulus induces the expression of C/EBPβ, which then activates the late-stage factors PPARγ and C/EBPα, leading to the formation of lipid droplets [35]. We investigated the effect of CypB’s PPIase activity on the expression of proteins associated with adipocyte differentiation. Protein levels of the early adipogenesis marker C/EBPβ were measured after two days of MDI treatment by Western blot analysis. As shown in Figure 1B, C/EBPβ expression was significantly diminished in CypB/R95A cells compared to pcDNA and CypB/WT cells. Similarly, after seven days of MDI treatment, the protein levels of late adipogenesis markers PPARγ and FABP4 were also markedly decreased in CypB/R95A cells (Figure 1C). This result indicates a significant reduction in the expression levels in CypB/R95A cells compared to both pcDNA and CypB/WT cells. However, the expression of C/EBPα in CypB/R95A cells was reduced compared to CypB/WT yet remained at levels comparable to those in pcDNA-transfected control cells.

### 3.2. Inhibition of CypB’s PPIase Activity Suppresses Mitotic Clonal Expansion

**Figure 2:**
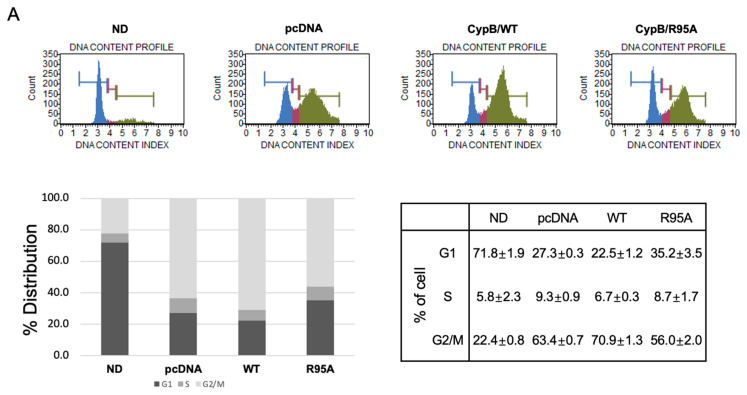

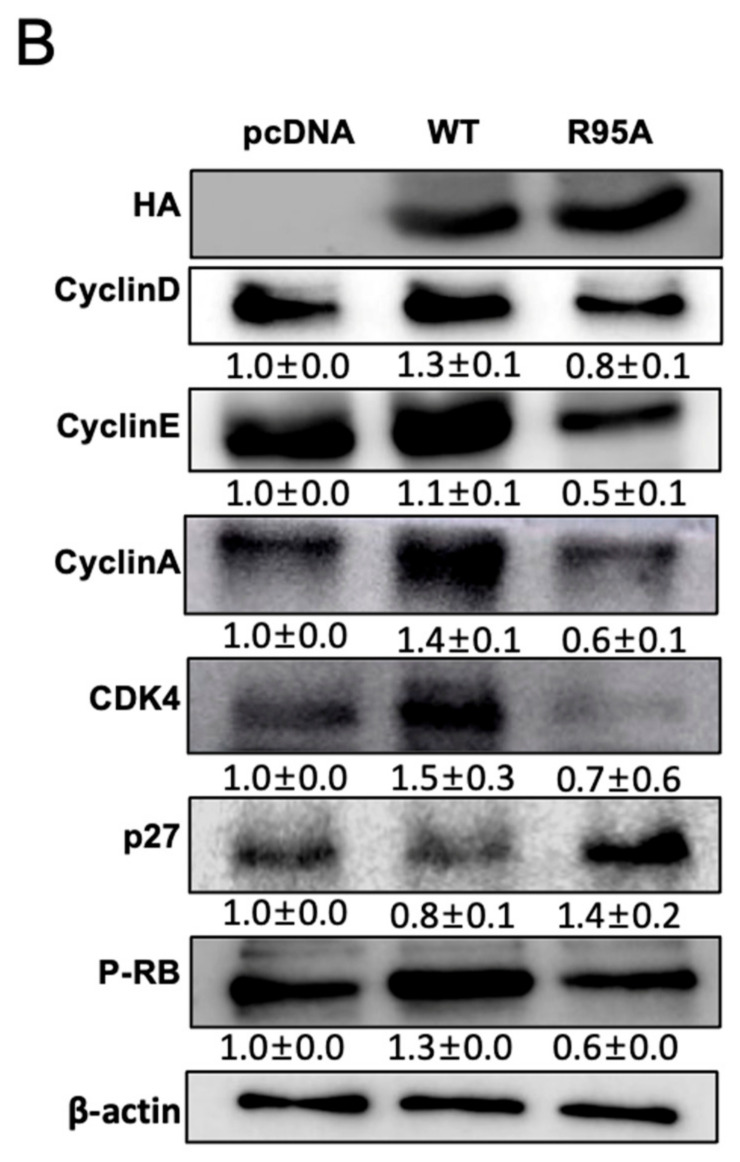
Effect of the PPIase activity of CypB on cell cycle progression during the differentiation of 3T3-L1 cells. 3T3-L1 cells at full confluence were treated with MDI for 24 h to prompt adipocyte differentiation. Post treatment, the cells were collected and fixed using 70% ethanol. The progression of the cell cycle was then assessed using the Muse™ Cell Analyzer. The cells were stained according to the specified protocol with the Muse™ Cell Cycle Kit. (**A**) The distribution of cells across the different stages of the cell cycle (G1, S, and G2/M) was determined by analyzing the cellular DNA content with the Muse™ Cell Analyzer, with the results expressed as a percentage for each phase. In the graph, blue represents the G1/G0 phase, red represents the S phase, and green represents the G2/M phase. (**B**) The protein expression levels of cyclin D, cyclin E, cyclin A, CDK4, p27, and pRB were evaluated via Western blot, with vinculin serving as the loading control. The presented data represent the average ± standard deviation from six independent experiments.

During the early stages of adipocyte differentiation, mitotic clonal expansion occurs, during which the previously arrested cell cycle resumes. To investigate the impact of CypB’s PPIase activity on this process, confluent 3T3-L1 cells were treated with the adipogenic inducer MDI for 24 h, and cell cycle changes were analyzed using a Muse™ Cell Analyzer (Figure 2A). As illustrated, the population of the G2/M phase decreased in CypB/R95A cells compared to CypB/WT cells during adipocyte differentiation. The cell DNA content in the G1 phase for pcDNA, CypB/WT, and CypB/R95A was 27.3%, 22.5%, and 35.2%, respectively, and in the G2/M phase, it was 63.4%, 70.9%, and 56.0%, respectively. Next, to determine the effect of PPIase activity inhibition on the expression of cell cycle-related proteins, we examined the protein levels of Cyclin D, Cyclin E, Cyclin A, CDK4, p27, and p-RB (Figure 2B). The expression of p27, which inhibits the Cyclin E/CDK2 complex, was observed to be increased in CypB/R95A cells compared to pcDNA and CypB/WT cells. Conversely, the levels of Cyclin D, Cyclin E, Cyclin A, and p-RB were found to be markedly reduced in CypB/R95A cells. These results suggest that inhibition of PPIase activity leads to the decreased phosphorylation of Rb, thereby hindering progression to the S phase and inducing G1 arrest.

### 3.3. Inhibition of CypB’s PPIase Activity Suppresses of AKT/mTOR Signaling Pathway in 3T3-L1 Cells

**Figure 3:**
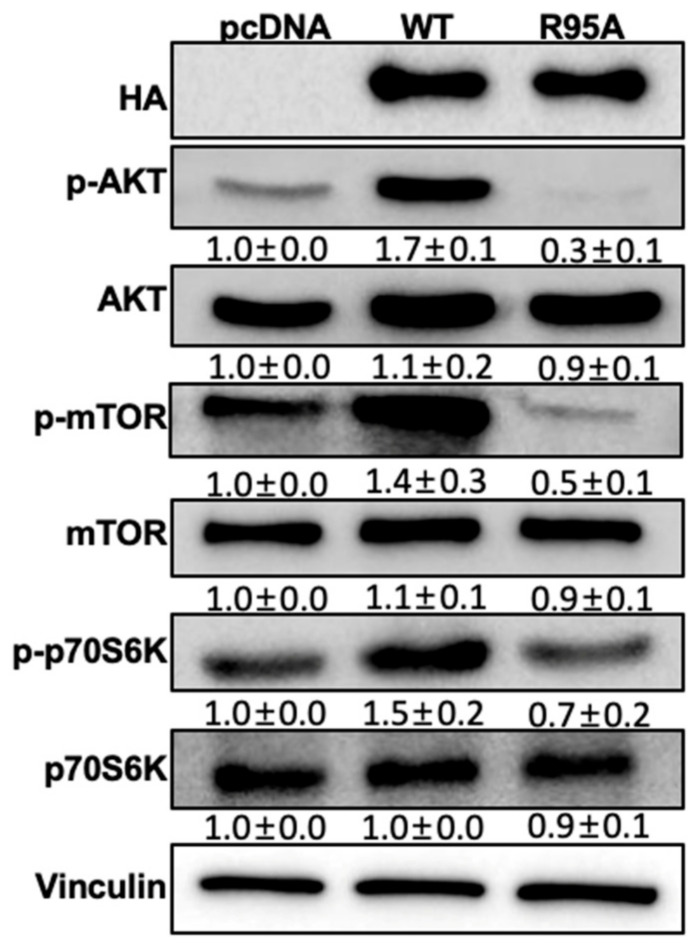
Effect of the PPIase activity of CypB on AKT/mTOR signaling pathway during the differentiation of 3T3-L1 cells. The cells were induced to differentiate into adipocytes with a 24 h MDI treatment. The molecules involved in the AKT/mTOR signaling pathway, specifically p-AKT, p-mTOR, and p-p70S6K, were observed in both CypB/WT and CypB/R95A through Western blotting. The protein band intensities were measured using ImageJ software and the values were adjusted based on vinculin expression. The results are expressed as the mean ± standard deviation from six individual experiments.

The AKT/mTOR pathway influences lipid metabolism via the insulin pathway, playing a crucial role in the differentiation of adipocyte [36]. In this study, we sought to investigate the effect of the PPIase activity of CypB on the AKT/mTOR signaling pathway during the differentiation of adipocytes (Figure 3). CypB/WT activated the AKT/mTOR pathway, resulting in the elevated expression of p-AKT and p-mTOR. Conversely, CypB/R95A inhibited the expression of p-AKT and p-mTOR. P70S6 kinase (P70S6K) is a serine/threonine protein kinase that is activated by mitogens and plays a crucial role in regulating cell growth and progression through the G1 cell cycle. While CypB/WT increased the expression of p-P70S6K, CypB/R95A decreased its protein levels. These findings indicate that CypB’s PPIase activity is a critical component of the AKT/mTOR pathway, which is involved in the differentiation of adipocytes.

### 3.4. The PPIase Activity of CypB Affects Lipid Metabolism via the XBP1s Pathway

**Figure 4:**
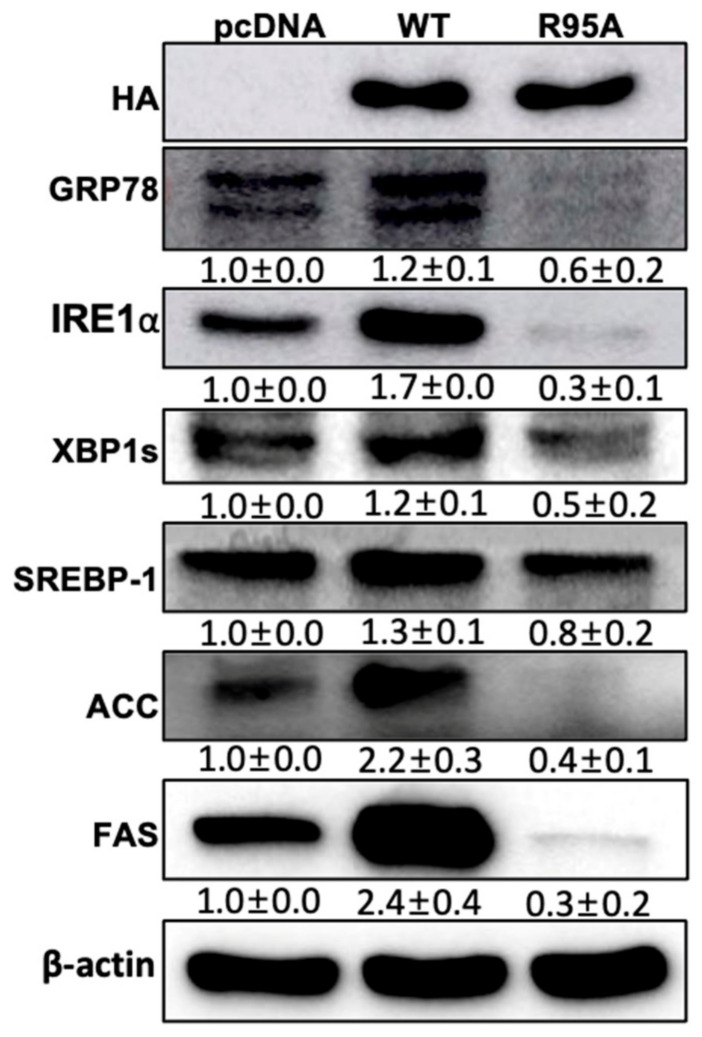
The PPIase activity of CypB affects lipid metabolism via the XBP1s pathway. After initiating adipogenesis and waiting for seven days, 3T3-L1 cells that had reached full confluence were analyzed using Western blot to assess the levels of GRP78, IRE1α-XBP1s, and key lipogenic indicators such as XBP1s, SREBP-1, ACC, and FAS. The protein band intensities were measured with ImageJ software and normalized against β-actin expression. The results are shown as the average ± standard deviation from six individual experiments.

A critical regulator of hepatic lipid metabolism homeostasis is the IRE1α-XBP1 pathway. IRE1α-deficient mice exhibit increased steatosis and decreased plasma lipid levels due to the decreased expression of C/EBPβ, C/EBPδ, PPARγ, and triglyceride synthesis enzymes [37]. Additionally, IRE1α and Xbp1 mRNA are known to be essential for cell type differentiation and function [38]. Separately, it has been established that the IRE1α-XBP1 signaling pathway activates lipogenesis in the liver [39]. Based on these findings, we examined the effect of CypB’s PPIase activity on IRE1α-XBP1-mediated lipogenesis (Figure 4). The ER stress marker GRP78 was found to be decreased in CypB/R95A, compared to pcDNA and CypB/WT. Subsequently, the expression of IRE1α and XBP1, key components of the UPR in response to ER stress, was assessed and found to be similarly reduced. Moreover, the expression of lipogenesis markers SREBP1, ACC, and FAS was also diminished in CypB/R95A, indicating that CypB’s PPIase activity may influence the XBP1s pathway.

## 4. Discussion

PPIase is an enzyme that regulates the folding of proline-based proteins within cells. This folding process significantly influences the secondary and tertiary structure formation of proteins, affecting their maturation, stability, and interactions. PPIase catalyzes the interconversion of the cis-1-isomer to the trans-1-isomer by speeding the rotation around the imidic peptide bond preceding the proline residues. Among the PPIase family, FK506-binding proteins (FKBPs) and Pin1 are notable members. FKBP51, one of the FK506-binding proteins, and Pin1 have been reported to affect adipogenesis. In addition, our previous study demonstrated the importance of the Cyclophilin family member CypB in adipogenesis. Therefore, the aim of the present study was to investigate whether the presence or absence of PPIase activity has an effect on adipogenesis by comparing the CypB mutants, CypB/R95A, and CypB/WT. CypB’s PPIase activity enhances the adipogenesis initial factor C/EBPβ, subsequently influencing adipogenesis late factors such as FABP4, C/EBPα, and PPARγ, indicating its crucial role in adipogenesis.

**Figure 5:**
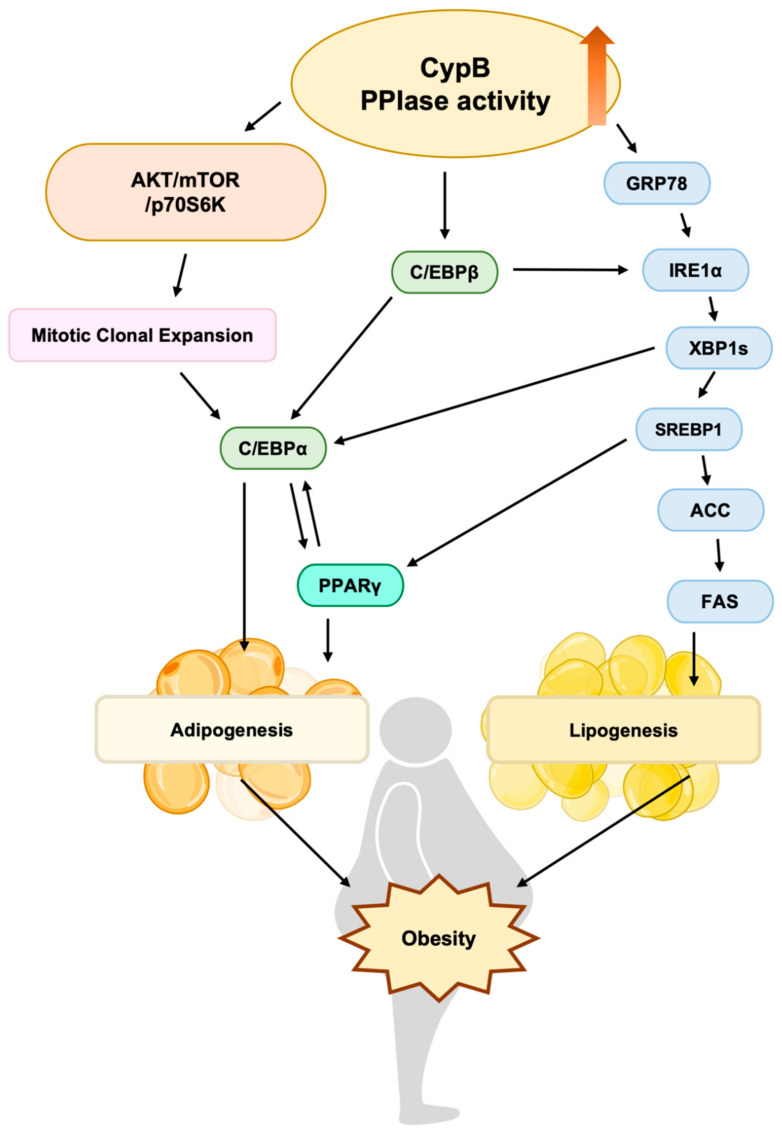
The diagram provided outlines how CypB’s PPIase activity governs the process of adipogenesis. This activity of CypB stimulates the AKT/mTOR signaling route, which results in mitotic clonal expansion. It also aids in triggering transcription factors that play a role in adipogenesis and enhances the function of IRE1α-XBP1, which in turn activates factors involved in lipogenesis. These intricate and diverse processes collectively play a part in the development of obesity.

The AKT/mTOR signaling pathway plays a key role in cell proliferation and in the development of adipocytes [40]. Our preceding findings indicated that CypB exerts control over the differentiation of adipocytes via the AKT/mTOR/p70S6K signaling cascade [10]. Specifically, it is established that the mammalian target of rapamycin (mTOR), via its downstream kinase, p70S6K, plays a pivotal role in regulating the progression of the cell cycle [40]. This study elucidated the impact of inhibiting CypB’s PPIase activity on lipid metabolism related to the AKT/mTOR pathway. Our findings indicate that the defective PPIase activity of CypB results in the attenuation of the phosphorylation of mTOR and its targets downstream partner, p70S6K (Figure 5). This implies that the inhibition of the mTOR pathway by defective CypB’s PPIase activity suppresses lipid accumulation.

Prior studies have indicated that the levels of the p27 protein diminish during the phase transition of mitotic clonal expansion in 3T3-L1 cells through the action of the 26S proteasome [10]. This investigation consistently demonstrated a reduction in p27 expression levels as a result of CypB/WT. However, in CypB/R95A cells, elevated levels of p27 were observed, which inhibited CDK2/Cyclin E, CDK2/Cyclin A, and CDK4/Cyclin D complexes, resulting in the reduced levels of phosphorylated Rb (p-RB). Consequently, the phosphorylation of Rb in subsequent stages was inhibited, which likely caused a blockade in the progression to the S phase and induced G1 arrest. The observed alterations in the expression patterns of proteins involved in the G1/S transition indicate that the induction of G1 arrest in 3T3-L1 cells results in the inhibition of cell proliferation, thereby leading to the suppression of mitotic clonal expansion.

Numerous lipogenic enzymes responsible for the synthesis of triglycerides (TGs) and fatty acids are involved in lipid accumulation during adipogenesis. One such lipogenic pathway is the IRE1α-XBP1 pathway. Among these, IRE1α, under conditions of ER stress, influences the expression of C/EBPβ, C/EBPδ, PPARγ, and key enzymes required for TG biosynthesis [41]. Recent studies have indicated that C/EBPβ can stimulate Xbp1 mRNA expression, and spliced XBP1 directly promotes C/EBPα expression during adipogenesis [37]. Furthermore, inducible liver-specific deletion of XBP1 resulted in marked hypocholesterolemia and hypotriglyceridemia due to decreased hepatic lipid production [42]. Additionally, the overexpression of the activated form of XBP1s has been shown to enhance promoter activity of SREBP-1 and FAS genes [39].

This study demonstrates that the PPIase activity of CypB affects lipid metabolism through the XBP1s pathway. CypB/R95A, in comparison to CypB/WT, resulted in the suppression of IRE1α-XBP1 protein expression, which in turn inhibited lipogenesis-related genes (SREBP1, FAS, ACC) (Figure 5). These results demonstrate that CypB PPIase activity is critical for adipocyte differentiation and obesogenic pathways. Specifically, the inhibition of CypB’s PPIase activity can regulate adipocyte differentiation and lipid metabolism, providing a foundational basis for developing new therapeutic strategies for obesity. This research will improve our understanding of adipocyte differentiation and obesity-related signaling pathways and contribute to the development of effective approaches for the prevention and adjuvant treatment of obesity. However, as this study is based on in vitro experimental results, further in-depth validation through human or animal studies is required. To substantiate the potential of CypB PPIase activity in the treatment of obesity, we propose several future research directions. First, in vivo studies using obesity animal models are essential to evaluate the systemic effects of CypB PPIase activity on adipogenesis, inflammation, and metabolic regulation. Additionally, clinical trials are necessary to further validate these findings in human subjects. Furthermore, exploring the possibility of combining CypB PPIase inhibitors with existing treatment modalities could enhance efficacy and reduce the required dosages of other medications. These studies will be critical in developing effective and targeted therapeutic strategies for obesity management.

## 5. Conclusions

Our study provides clear evidence of the critical role of PPIase activity of CypB in adipogenesis and lipid metabolism, underscoring its significance for obesity treatment and prevention. Specifically, inhibiting CypB’s PPIase activity can effectively suppress adipocyte formation, thereby promoting reduction in adipose tissue in obese individuals. Furthermore, CypB’s PPIase activity affects lipid metabolism through key pathways including AKT/mTOR and IRE1α-XBP1, helping to prevent and treat obesity-related metabolic disorders such as diabetes and hyperlipidemia. Additionally, by regulating inflammation-related proteins, CypB’s PPIase activity holds potential for ameliorating chronic inflammatory conditions linked to obesity, thereby enhancing overall health outcomes in obese patients. Moreover, the development of novel therapies targeting CypB’s PPIase activity offers a promising strategy to complement existing obesity treatments, potentially providing diversified therapeutic options tailored to individual patient needs. These findings highlight the multifaceted implications of our research for the effective management of obesity-related health challenges.

## Data Availability

The original contributions presented in the study are included in the article, further inquiries can be directed to the corresponding author.
